# Publisher Correction: An Archimedes’ screw for light

**DOI:** 10.1038/s41467-022-32352-7

**Published:** 2022-08-03

**Authors:** Emanuele Galiffi, Paloma A. Huidobro, J. B. Pendry

**Affiliations:** 1 Advanced Science Research Center, City University of New York, 85 St. Nicholas Terrace, New York, NY 10031 USA; 2 Instituto de Telecomunicações, Instituto Superior Tecnico-University of Lisbon, Avenida Rovisco Pais 1, 1049-001 Lisboa, Portugal; 3 The Blackett Laboratory, Department of Physics, Imperial College London, South Kensington Campus, London, SW7 2AZ UK

**Keywords:** Metamaterials, Nanophotonics and plasmonics, Photonic crystals

Correction to: *Nature Communications* 10.1038/s41467-022-30079-z, published online 09 May 2022

The PDF version of this article contained an error in equation 12. The M matrix in equation 12 was incorrectly formatted as a row vector where they should form a square matrix.

This has to be corrected only in the PDF version of the article, the HTML version is Ok.

How it appears now:

$${\omega }_{n}\left(\begin{array}{c}{{{{\bf{F}}}}}_{n}^{\rightharpoonup }\\ {{{{\bf{F}}}}}_{n}^{\leftharpoonup }\end{array}\right)=({{\mathbb{M}}}_{{\rightharpoonup }_{,n}}^{\rightharpoonup }{{\mathbb{M}}}_{{\leftharpoonup }_{,n}}^{\rightharpoonup }{{\mathbb{M}}}_{{\rightharpoonup }_{,n}}^{\leftharpoonup }{{\mathbb{M}}}_{{\leftharpoonup }_{,n}}^{\leftharpoonup })\left(\begin{array}{c}{F}_{n}^{\rightharpoonup }\\ {{{{\bf{F}}}}}_{n}^{\leftharpoonup }\end{array}\right)$$

How it should appear:

$${\omega }_{n}\left(\begin{array}{c}{{{{\bf{F}}}}}_{n}^{\rightharpoonup }\\ {{{{\bf{F}}}}}_{n}^{\leftharpoonup }\end{array}\right)=\left(\begin{array}{cc}{{\mathbb{M}}}_{{\rightharpoonup }_{,n}}^{\rightharpoonup } & {{\mathbb{M}}}_{{\leftharpoonup }_{,n}}^{\rightharpoonup }\\ {{\mathbb{M}}}_{{\rightharpoonup }_{,n}}^{\leftharpoonup } & {{\mathbb{M}}}_{{\leftharpoonup }_{,n}}^{\leftharpoonup }\end{array}\right)\left(\begin{array}{c}{{{{\bf{F}}}}}_{n}^{\rightharpoonup }\\ {{{{\bf{F}}}}}_{n}^{\leftharpoonup }\end{array}\right)$$

