# Supplementary material for: An Archimedes' screw for light
Source: Nat Commun. 2022 May 9;13:2523. doi: 10.1038/s41467-022-30079-z (PMC9085788; doi:10.1038/s41467-022-30079-z)
Supplement: Supplementary file 1 — Supplementary Information [file 41467_2022_30079_MOESM1_ESM.pdf]

An Archimedes' Screw for Light - Supplementary Information

Emanuele Galiffi, Paloma A. Huidobro and J. B. Pendry

April 8, 2022

Supplementary Discussion - Transmission for ε-only modulations

Although the assumption of impedance-matching simplifies the analytics further, it is not a necessary assumption for the realization of these instabilities, as we show in Fig. 1 for the case of $\alpha_\varepsilon = 0.4$, $\alpha_\mu = 0$ and $\Omega = 0.8$. For the right-hand polarized case, the dynamics is equivalent to that shown in the main manuscript, whereas for the LHP one we simply observe beating. Note that back-scattering is now allowed due to the relaxation of the impedance-matching condition, leading to the additional beating observe in the LHP case.

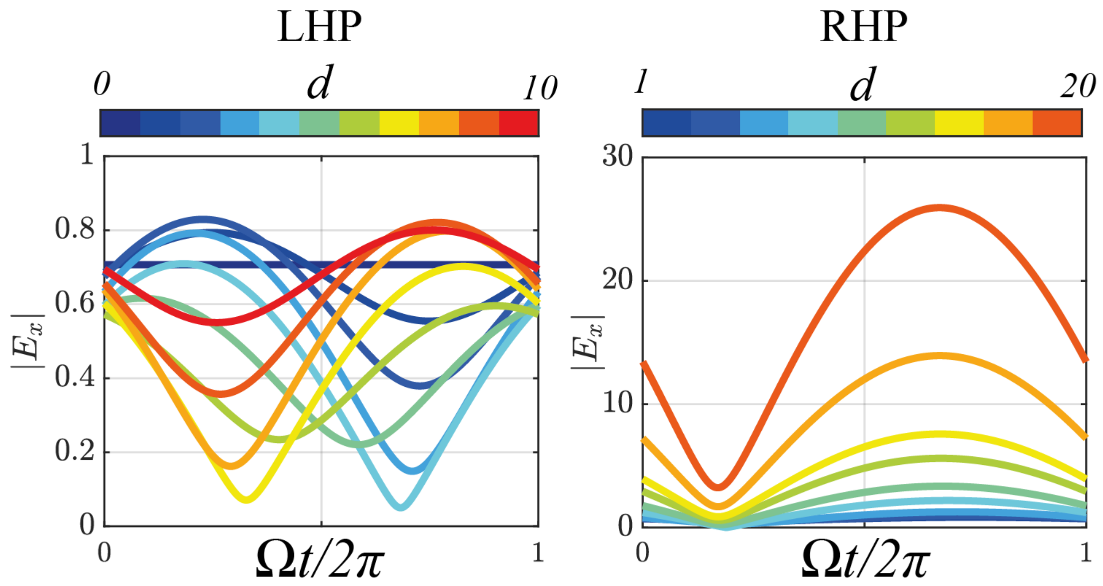

Supplementary Figure 1: Transmitted intensity as a function of time for the case where only epsilon is modulated. The left panel shows the case of a LHP incident wave and the right panel that of a RHP one.

Supplementary Note 1 - Analytic solution for D and B for $\omega(k)$

Let us consider a medium with a uniaxial perturbation of its electromagnetic tensors, which rotates describing a helix in space and time. The tensors are subject the following coordinate transformation:

$$\varepsilon/\varepsilon_1 = \begin{pmatrix}1&0&0\\0&1&0\\0&0&1\end{pmatrix} + \begin{pmatrix}c(\theta^-)&-s(\theta^-)&0\\s(\theta^-)&c(\theta^-)&0\\0&0&1\end{pmatrix}\begin{pmatrix}2\alpha_\varepsilon&0&0\\0&0&0\\0&0&0\end{pmatrix}\begin{pmatrix}c(\theta^-)&s(\theta^-)&0\\-s(\theta^-)&c(\theta^-)&0\\0&0&1\end{pmatrix}, \quad (1)$$

and similarly for μ:

$$\mu/\mu_1 = \begin{pmatrix}1&0&0\\0&1&0\\0&0&1\end{pmatrix} + \begin{pmatrix}c(\theta^+)&-s(\theta^+)&0\\s(\theta^+)&c(\theta^+)&0\\0&0&1\end{pmatrix}\begin{pmatrix}0&0&0\\0&2\alpha_\mu&0\\0&0&0\end{pmatrix}\begin{pmatrix}c(\theta^+)&s(\theta^+)&0\\-s(\theta^+)&c(\theta^+)&0\\0&0&1\end{pmatrix}, \quad (2)$$

where $\theta^\pm = gz - \Omega t \pm \phi$ and ϵ_1 and μ_1 are the background permittivity and permeability, and we denote, for brevity, cosines and sines as c and s respectively. The constant ϕ results in a dephasing of 2ϕ between the electric and the magnetic components of the modulation. Note that the modulations of the $x-x$ component of ε and the $y-y$ of μ coincide, so that the modulation acts on a specific linear polarisation wherever at a specific point in spacetime (e.g.

for $\theta = \phi = 0$, only x-polarised waves experience a different refractive index, while y-polarised waves are unaffected). Performing the matrix multiplications, we obtain the following forms for the modulated tensors:

$$\varepsilon/\varepsilon_1 = \begin{pmatrix}1 & 0 & 0\\ 0 & 1 & 0\\ 0 & 0 & 1\end{pmatrix} + 2\alpha_\varepsilon \begin{pmatrix} c^2(\theta^-) & c(\theta^-)s(\theta^-) & 0\\ c(\theta^-)s(\theta^-) & s^2(\theta^-) & 0\\ 0 & 0 & 0\end{pmatrix} \tag{3}$$

$$\mu/\mu_1 = \begin{pmatrix}1 & 0 & 0\\ 0 & 1 & 0\\ 0 & 0 & 1\end{pmatrix} + 2\alpha_\mu \begin{pmatrix} s^2(\theta^+) & -c(\theta^+)s(\theta^+) & 0\\ -c(\theta^+)s(\theta^+) & c^2(\theta^+) & 0\\ 0 & 0 & 0\end{pmatrix} \tag{4}$$

Let us start from the complete Maxwell Equations for a non-dispersive medium, with $\mathbf{D} = \hat{\varepsilon}\mathbf{E}$ and $\mathbf{B} = \hat{\mu}\mathbf{H}$ (where we have already inverted the electromagnetic tensors to express $\mathbf{E}$ as $\hat{\varepsilon}^{-1}\mathbf{D}$ and $\mathbf{H}$ as $\hat{\mu}^{-1}\mathbf{H}$). In this instance, we shall restrict ourselves to the case of normal incidence, so that $\frac{\partial}{\partial x} = \frac{\partial}{\partial y} = 0$.

$$\frac{\partial D_y}{\partial t} = \frac{1}{\mu_1}\left\{ -2\bar{\alpha}_\mu g[-s(2\theta^+)B_x + c(2\theta^+)B_y] + [1 + 2\bar{\alpha}_\mu s^2(\theta^+)]\frac{\partial B_x}{\partial z} - 2\bar{\alpha}_\mu c(\theta^+)s(\theta^+)\frac{\partial B_y}{\partial z}\right\} \tag{5}$$

$$\frac{\partial D_x}{\partial t} = \frac{1}{\mu_1}\left\{ +2\bar{\alpha}_\mu g[+c(2\theta^+)B_x + s(2\theta^+)B_y] - [1 + 2\bar{\alpha}_\mu c^2(\theta^+)]\frac{\partial B_y}{\partial z} + 2\bar{\alpha}_\mu c(\theta^+)s(\theta^+)\frac{\partial B_x}{\partial z}\right\} \tag{6}$$

$$\frac{\partial B_y}{\partial t} = \frac{1}{\varepsilon_1}\left\{ -2\bar{\alpha}_\varepsilon g[-s(2\theta^-)D_x + c(2\theta^-)D_y] - [1 + 2\bar{\alpha}_\varepsilon c^2(\theta^-)]\frac{\partial D_x}{\partial z} - 2\bar{\alpha}_\varepsilon c(\theta^-)s(\theta^-)\frac{\partial D_y}{\partial z}\right\} \tag{7}$$

$$\frac{\partial B_x}{\partial t} = \frac{1}{\varepsilon_1}\left\{ +2\bar{\alpha}_\varepsilon g[+c(2\theta^-)D_x + s(2\theta^-)D_y] + [1 + 2\bar{\alpha}_\varepsilon s^2(\theta^-)]\frac{\partial D_y}{\partial z} + 2\bar{\alpha}_\varepsilon c(\theta^-)s(\theta^-)\frac{\partial D_x}{\partial z}\right\} \tag{8}$$

where we defined $\bar{\alpha}_\varepsilon = -\frac{\alpha_\varepsilon}{1+2\alpha_\varepsilon}$ and $\bar{\alpha}_\mu = -\frac{\alpha_\mu}{1+2\alpha_\mu}$. We can rewrite this as:

$$\mu_1\frac{\partial D_y}{\partial t} = +\frac{\partial B_x}{\partial z} + 2\bar{\alpha}_\mu g B_y - 4\bar{\alpha}_\mu g c(\theta^+)[-s(\theta^+)B_x + c(\theta^+)B_y] - 2\bar{\alpha}_\mu s(\theta^+)[-s(\theta^+)\frac{\partial B_x}{\partial z} + c(\theta^+)\frac{\partial B_y}{\partial z}] \tag{9}$$

$$\mu_1\frac{\partial D_x}{\partial t} = -\frac{\partial B_y}{\partial z} + 2\bar{\alpha}_\mu g B_x + 4\bar{\alpha}_\mu g s(\theta^+)[-s(\theta^+)B_x + c(\theta^+)B_y] - 2\bar{\alpha}_\mu c(\theta^+)[-s(\theta^+)\frac{\partial B_x}{\partial z} + c(\theta^+)\frac{\partial B_y}{\partial z}] \tag{10}$$

$$\varepsilon_1\frac{\partial B_y}{\partial t} = -\frac{\partial D_x}{\partial z} - 2\bar{\alpha}_\varepsilon g D_y + 4\bar{\alpha}_\varepsilon g s(\theta^-)[+c(\theta^-)D_x + s(\theta^-)D_y] - 2\bar{\alpha}_\varepsilon c(\theta^-)[+c(\theta^-)\frac{\partial D_x}{\partial z} + s(\theta^-)\frac{\partial D_y}{\partial z}] \tag{11}$$

$$\varepsilon_1\frac{\partial B_x}{\partial t} = +\frac{\partial D_y}{\partial z} - 2\bar{\alpha}_\varepsilon g D_x + 4\bar{\alpha}_\varepsilon g c(\theta^-)[+c(\theta^-)D_x + s(\theta^-)D_y] + 2\bar{\alpha}_\varepsilon s(\theta^-)[+c(\theta^-)\frac{\partial D_x}{\partial z} + s(\theta^-)\frac{\partial D_y}{\partial z}] \tag{12}$$

We now combine the above equations as follows:

$$s(\theta^+)Eq.(5) + c(\theta^+)Eq.(6) \qquad\qquad c(\theta^+)Eq.(5) - s(\theta^+)Eq.(6) \tag{13}$$

$$s(\theta^-)Eq.(7) + c(\theta^-)Eq.(8) \qquad\qquad c(\theta^-)Eq.(7) - s(\theta^-)Eq.(8) \tag{14}$$

and define the screwing coordinates:

$$x'(\theta) = c(\theta)x + s(\theta)y \qquad\qquad y'(\theta) = -s(\theta)x + c(\theta)y \tag{15}$$

as well as the related screwing field components:

$$D^+_{x'}(\theta^+) = c(\theta^+)D_x(\theta^+) + s(\theta^+)D_y(\theta^+) \qquad\qquad D_{y'}(\theta^+) = -s(\theta^+)D_x(\theta^+) + c(\theta^+)D_y(\theta^+) \tag{16}$$

$$B^-_{x'}(\theta) = c(\theta^-)B_x(\theta^-) + s(\theta^-)B_y(\theta^-) \qquad\qquad B^-_{y'}(\theta^-) = -s(\theta^-)B_x(\theta^-) + c(\theta^-)B_y(\theta^-) \tag{17}$$

where we need to account for the space-and time-dependence of $\theta^\pm(z,t)$ as we include the trigonometric functions within the derivatives via the chain-rule:

$$c(\theta)\frac{\partial\psi}{\partial z} = \frac{\partial(c(\theta)\psi)}{\partial z} + gs(\theta)\psi \qquad\qquad s(\theta)\frac{\partial\psi}{\partial z} = \frac{\partial(s(\theta)\psi)}{\partial z} - gc(\theta)\psi \tag{18}$$

$$c(\theta)\frac{\partial\psi}{\partial t} = \frac{\partial(c(\theta)\psi)}{\partial t} - \Omega s(\theta)\psi \qquad\qquad s(\theta)\frac{\partial\psi}{\partial t} = \frac{\partial(s(\theta)\psi)}{\partial t} + \Omega c(\theta)\psi, \tag{19}$$

we thus arrive at the new set of equations:

$$\frac{\partial D^+_{x'}}{\partial t} + \Omega D^+_{y'} = \frac{1}{\mu_1}\Big\{ - \frac{\partial B^+_{y'}}{\partial z} - gB^+_{x'} - 2\bar{\alpha}_\mu \frac{\partial B^+_{y'}}{\partial z} + [-s(\theta^+)\frac{\partial}{\partial x} + c(\theta^+)\frac{\partial}{\partial y}]B_z \Big\} \quad (20)$$

$$\frac{\partial D^+_{y'}}{\partial t} - \Omega D^+_{x'} = \frac{1}{\mu_1}\Big\{ + \frac{\partial B^+_{x'}}{\partial z} - gB^+_{y'} - 2\bar{\alpha}_\mu g B^+_{y'} - [+c(\theta^+)\frac{\partial}{\partial x} + s(\theta^+)\frac{\partial}{\partial y}]B_z \Big\} \quad (21)$$

$$\frac{\partial B^-_{x'}}{\partial t} + \Omega B^-_{y'} = \frac{1}{\mu_1}\Big\{ + \frac{\partial D^-_{y'}}{\partial z} + gD^-_{x'} + 2\bar{\alpha}_\varepsilon g D^-_{x'} - [-s(\theta^-)\frac{\partial}{\partial x} + c(\theta^-)\frac{\partial}{\partial y}]D_z \Big\} \quad (22)$$

$$\frac{\partial B^-_{y'}}{\partial t} - \Omega B^-_{x'} = \frac{1}{\mu_1}\Big\{ - \frac{\partial D^-_{x'}}{\partial z} + gD^-_{y'} - 2\bar{\alpha}_\varepsilon \frac{\partial D^-_{x'}}{\partial z} + [+c(\theta^-)\frac{\partial}{\partial x} + s(\theta^-)\frac{\partial}{\partial y}]D_z \Big\} \quad (23)$$

We now want to move to a basis of forward and backward-propagating waves. After taking the combinations:

$$Eq.20 + \sqrt{\frac{\varepsilon_1}{\mu_1}} Eq.23 \qquad\qquad Eq.20 - \sqrt{\frac{\varepsilon_1}{\mu_1}} Eq.23 \quad (24)$$

$$Eq.21 + \sqrt{\frac{\varepsilon_1}{\mu_1}} Eq.22 \qquad\qquad Eq.21 - \sqrt{\frac{\varepsilon_1}{\mu_1}} Eq.22, \quad (25)$$

rescaling the B fields as $\bar{B} = \sqrt{\varepsilon_1/\mu_1} B$, and defining the sums and differences between the electric and the magnetic modulations $\bar{\alpha}^\pm = \bar{\alpha}_\varepsilon \pm \bar{\alpha}_\mu$ we can use the identities:

$$\bar{\alpha}_\varepsilon D_i + \bar{\alpha}_\mu \bar{B}_j = \frac{1}{2}[(\bar{\alpha}_\varepsilon + \bar{\alpha}_\mu)(D_i + \bar{B}_j) + (\bar{\alpha}_\varepsilon - \bar{\alpha}_\mu)(D_i - \bar{B}_j)] = \frac{1}{2}[\bar{\alpha}^+(D_i + \bar{B}_j) + \bar{\alpha}^-(D_i - \bar{B}_j)] \quad (26)$$

$$\bar{\alpha}_\varepsilon D_i - \bar{\alpha}_\mu \bar{B}_j = \frac{1}{2}[(\bar{\alpha}_\varepsilon - \bar{\alpha}_\mu)(D_i + \bar{B}_j) + (\bar{\alpha}_\varepsilon + \bar{\alpha}_\mu)(D_i - \bar{B}_j)] = \frac{1}{2}[\bar{\alpha}^-(D_i + \bar{B}_j) + \bar{\alpha}^+(D_i - \bar{B}_j)] \quad (27)$$

to obtain:

$$\frac{\partial}{\partial t}(D^+_{x'} + \bar{B}^-_{y'}) + \Omega(D^+_{y'} - \bar{B}^-_{x'}) = c_1\Big\{ - \frac{\partial}{\partial z}(D^-_{x'} + \bar{B}^+_{y'}) + g(D^-_{y'} - \bar{B}^+_{x'}) - \frac{\partial}{\partial z}\Big[\bar{\alpha}^+(D^-_{x'} + \bar{B}^+_{y'}) + \bar{\alpha}^-(D^-_{x'} - \bar{B}^+_{y'})\Big]\Big\} \quad (28)$$

$$\frac{\partial}{\partial t}(D^+_{x'} - \bar{B}^-_{y'}) + \Omega(D^+_{y'} + \bar{B}^-_{x'}) = c_1\Big\{ + \frac{\partial}{\partial z}(D^-_{x'} - \bar{B}^+_{y'}) - g(D^-_{y'} + \bar{B}^+_{x'}) + \frac{\partial}{\partial z}\Big[\bar{\alpha}^-(D^-_{x'} + \bar{B}^+_{y'}) + \bar{\alpha}^+(D^-_{x'} - \bar{B}^+_{y'})\Big]\Big\} \quad (29)$$

$$\frac{\partial}{\partial t}(D^+_{y'} + \bar{B}^-_{x'}) - \Omega(D^+_{x'} - \bar{B}^-_{y'}) = c_1\Big\{ + \frac{\partial}{\partial z}(D^-_{y'} + \bar{B}^+_{x'}) + g(D^-_{x'} - \bar{B}^+_{y'}) + g[\bar{\alpha}^-(D^-_{x'} + \bar{B}^+_{y'}) + \bar{\alpha}^+(D^-_{x'} - \bar{B}^+_{y'})]\Big\} \quad (30)$$

$$\frac{\partial}{\partial t}(D^+_{y'} - \bar{B}^-_{x'}) - \Omega(D^+_{x'} + \bar{B}^-_{y'}) = c_1\Big\{ - \frac{\partial}{\partial z}(D^-_{y'} - \bar{B}^+_{x'}) - g(D^-_{x'} + \bar{B}^+_{y'}) - g[\bar{\alpha}^+(D^-_{x'} + \bar{B}^+_{y'}) + \bar{\alpha}^-(D^-_{x'} - \bar{B}^+_{y'})]\Big\} \quad (31)$$

Which allowed us to successfully remove all the θ-dependent terms from the equations. We are now left with 8 combinations of fields (due to the presence of both $\pm\phi$ terms), but only 4 equations. In order to reduce the number of variables, we can recognise that the individual terms above can be expanded as:

$$D^+_{x'} + \bar{B}^-_{y'} = [c(\theta^+)(+D_x) + c(\theta^-)(+\bar{B}_y)] + [s(\theta^+)(+D_y) + s(\theta^-)(-\bar{B}_x)] \quad (32)$$

$$D^+_{x'} - \bar{B}^-_{y'} = [c(\theta^+)(+D_x) + c(\theta^-)(-\bar{B}_y)] + [s(\theta^+)(+D_y) + s(\theta^-)(+\bar{B}_x)] \quad (33)$$

$$D^+_{y'} + \bar{B}^-_{x'} = [s(\theta^+)(-D_x) + s(\theta^-)(+\bar{B}_y)] + [c(\theta^+)(+D_y) + c(\theta^-)(+\bar{B}_x)] \quad (34)$$

$$D^+_{y'} - \bar{B}^-_{x'} = [s(\theta^+)(-D_x) + s(\theta^-)(-\bar{B}_y)] + [c(\theta^+)(+D_y) + c(\theta^-)(-\bar{B}_x)] \quad (35)$$

$$D^-_{x'} + \bar{B}^+_{y'} = [c(\theta^-)(+D_x) + c(\theta^+)(+\bar{B}_y)] + [s(\theta^-)(+D_y) + s(\theta^+)(-\bar{B}_x)] \quad (36)$$

$$D^-_{x'} - \bar{B}^+_{y'} = [c(\theta^-)(+D_x) + c(\theta^+)(-\bar{B}_y)] + [s(\theta^-)(+D_y) + s(\theta^+)(+\bar{B}_x)] \quad (37)$$

$$D^-_{y'} + \bar{B}^+_{x'} = [s(\theta^-)(-D_x) + s(\theta^+)(+\bar{B}_y)] + [c(\theta^-)(+D_y) + c(\theta^+)(-\bar{B}_x)] \quad (38)$$

$$D^-_{y'} - \bar{B}^+_{x'} = [s(\theta^-)(-D_x) + s(\theta^+)(-\bar{B}_y)] + [c(\theta^-)(+D_y) + c(\theta^+)(+\bar{B}_x)] \quad (39)$$

and use the relations:

$$c(\theta^+)\Psi + c(\theta^-)\Phi = \frac{1}{2}[(c(\theta^+) + c(\theta^-))(\Psi + \Phi) + (c(\theta^+) - c(\theta^-))(\Psi - \Phi)] = c(\theta)c(\phi)(\Psi + \Phi) - s(\theta)s(\phi)(\Psi - \Phi) \quad (40)$$

$$s(\theta^+)\Psi + s(\theta^-)\Phi = \frac{1}{2}[(s(\theta^+) + s(\theta^-))(\Psi + \Phi) + (s(\theta^+) - s(\theta^-))(\Psi - \Phi)] = s(\theta)c(\phi)(\Psi + \Phi) + c(\theta)s(\phi)(\Psi - \Phi), \quad (41)$$

which follow from the the trigonometric identities:

$$c(\theta^+) + c(\theta^-) = 2c(\theta)c(\phi) \qquad c(\theta^+) - c(\theta^-) = -2s(\theta)s(\phi) \tag{42}$$

$$s(\theta^+) + s(\theta^-) = 2s(\theta)c(\phi) \qquad s(\theta^+) - s(\theta^-) = +2c(\theta)s(\phi) \tag{43}$$

to write the equations above only as a function of the forward and backward-propagating fields:

$$F^{\rightharpoonup}_{x'} = c(\theta)(D_x + \bar{B}_y) + s(\theta)[D_y + (-\bar{B}_x)] \qquad F^{\rightharpoonup}_{y'} = -s(\theta)(D_x + \bar{B}_y) + c(\theta)[D_y + (-\bar{B}_x)] \tag{44}$$

$$F^{\leftharpoonup}_{x'} = c(\theta)(D_x - \bar{B}_y) + s(\theta)[D_y - (-\bar{B}_x)] \qquad F^{\leftharpoonup}_{y'} = -s(\theta)(D_x - \bar{B}_y) + c(\theta)[D_y - (-\bar{B}_x)] \tag{45}$$

so that:

$$D^+_{x'} + \bar{B}^-_{y'} = c(\phi)F^{\rightharpoonup}_{x'} + s(\phi)F^{\leftharpoonup}_{y'} \qquad D^-_{x'} + \bar{B}^+_{y'} = -s(\phi)F^{\leftharpoonup}_{y'} + c(\phi)F^{\rightharpoonup}_{x'} \tag{46}$$

$$D^+_{x'} - \bar{B}^-_{y'} = c(\phi)F^{\leftharpoonup}_{x'} + s(\phi)F^{\rightharpoonup}_{y'} \qquad D^-_{x'} - \bar{B}^+_{y'} = -s(\phi)F^{\rightharpoonup}_{y'} + c(\phi)F^{\leftharpoonup}_{x'} \tag{47}$$

$$D^+_{y'} + \bar{B}^-_{x'} = -s(\phi)F^{\rightharpoonup}_{x'} + c(\phi)F^{\leftharpoonup}_{y'} \qquad D^-_{y'} + \bar{B}^+_{x'} = c(\phi)F^{\leftharpoonup}_{y'} + s(\phi)F^{\rightharpoonup}_{x'} \tag{48}$$

$$D^+_{y'} - \bar{B}^-_{x'} = -s(\phi)F^{\leftharpoonup}_{x'} + c(\phi)F^{\rightharpoonup}_{y'} \qquad D^-_{y'} - \bar{B}^+_{x'} = c(\phi)F^{\rightharpoonup}_{y'} + s(\phi)F^{\leftharpoonup}_{x'} \tag{49}$$

Substituting the latter into Eqs. 28-31, we derive four coupled equations, which no longer depend on the spatiotemporal variable θ, which has been absorbed into our new set of basis functions. These can be cast into a standard eigenvalue problem by taking:

$$\begin{aligned}\frac{\partial}{\partial t}(c(\phi)F^{\rightharpoonup}_{x'} + s(\phi)F^{\leftharpoonup}_{y'}) = -\Omega(-s(\phi)F^{\leftharpoonup}_{x'} + c(\phi)F^{\rightharpoonup}_{y'}) + c_1\Big\{ &-\frac{\partial}{\partial z}(-s(\phi)F^{\leftharpoonup}_{y'} + c(\phi)F^{\rightharpoonup}_{x'}) + g(c(\phi)F^{\rightharpoonup}_{y'} + s(\phi)F^{\leftharpoonup}_{x'})\\ &-\frac{\partial}{\partial z}\Big[\bar{\alpha}^+(-s(\phi)F^{\leftharpoonup}_{y'} + c(\phi)F^{\rightharpoonup}_{x'}) + \bar{\alpha}^-(-s(\phi)F^{\rightharpoonup}_{y'} + c(\phi)F^{\leftharpoonup}_{x'})\Big]\Big\}\end{aligned} \tag{50}$$

$$\begin{aligned}\frac{\partial}{\partial t}(c(\phi)F^{\leftharpoonup}_{x'} + s(\phi)F^{\rightharpoonup}_{y'}) = -\Omega(-s(\phi)F^{\rightharpoonup}_{x'} + c(\phi)F^{\leftharpoonup}_{y'}) + c_1\Big\{ &+\frac{\partial}{\partial z}(-s(\phi)F^{\rightharpoonup}_{y'} + c(\phi)F^{\leftharpoonup}_{x'}) - g(c(\phi)F^{\leftharpoonup}_{y'} + s(\phi)F^{\rightharpoonup}_{x'})\\ &+\frac{\partial}{\partial z}\Big[\bar{\alpha}^-(-s(\phi)F^{\leftharpoonup}_{y'} + c(\phi)F^{\rightharpoonup}_{x'}) + \bar{\alpha}^+(-s(\phi)F^{\rightharpoonup}_{y'} + c(\phi)F^{\leftharpoonup}_{x'})\Big]\Big\}\end{aligned} \tag{51}$$

$$\begin{aligned}\frac{\partial}{\partial t}(-s(\phi)F^{\rightharpoonup}_{x'} + c(\phi)F^{\leftharpoonup}_{y'}) = +\Omega(c(\phi)F^{\leftharpoonup}_{x'} + s(\phi)F^{\rightharpoonup}_{y'}) + c_1\Big\{ &+\frac{\partial}{\partial z}(c(\phi)F^{\leftharpoonup}_{y'} + s(\phi)F^{\rightharpoonup}_{x'}) + g(-s(\phi)F^{\rightharpoonup}_{y'} + c(\phi)F^{\leftharpoonup}_{x'})\\ &+g[\bar{\alpha}^-(-s(\phi)F^{\leftharpoonup}_{y'} + c(\phi)F^{\rightharpoonup}_{x'}) + \bar{\alpha}^+(-s(\phi)F^{\rightharpoonup}_{y'} + c(\phi)F^{\leftharpoonup}_{x'})]\Big\}\end{aligned} \tag{52}$$

$$\begin{aligned}\frac{\partial}{\partial t}(-s(\phi)F^{\leftharpoonup}_{x'} + c(\phi)F^{\rightharpoonup}_{y'}) = +\Omega(c(\phi)F^{\rightharpoonup}_{x'} + s(\phi)F^{\leftharpoonup}_{y'}) + c_1\Big\{ &-\frac{\partial}{\partial z}(c(\phi)F^{\rightharpoonup}_{y'} + s(\phi)F^{\leftharpoonup}_{x'}) - g(-s(\phi)F^{\leftharpoonup}_{y'} + c(\phi)F^{\rightharpoonup}_{x'})\\ &-g[\bar{\alpha}^+(-s(\phi)F^{\leftharpoonup}_{y'} + c(\phi)F^{\rightharpoonup}_{x'}) + \bar{\alpha}^-(-s(\phi)F^{\rightharpoonup}_{y'} + c(\phi)F^{\leftharpoonup}_{x'})]\Big\}\end{aligned} \tag{53}$$

which we conveniently simplify by taking:

$$c(\phi)Eq.50 - s(\phi)Eq.52 \qquad s(\phi)Eq.51 + c(\phi)Eq.53 \qquad c(\phi)Eq.51 - s(\phi)Eq.53 \qquad s(\phi)Eq.50 + c(\phi)Eq.52 \tag{54}$$

leaving us with:

$$\begin{aligned}\frac{\partial F^{\rightharpoonup}_{x'}}{\partial t} = +(c_1 g - \Omega)F^{\rightharpoonup}_{y'} + c_1\Big\{ -\frac{\partial F^{\rightharpoonup}_{x'}}{\partial z} &- c(\phi)\frac{\partial}{\partial z}[\bar{\alpha}^+(-s(\phi)F^{\leftharpoonup}_{y'} + c(\phi)F^{\rightharpoonup}_{x'}) + \bar{\alpha}^-(-s(\phi)F^{\rightharpoonup}_{y'} + c(\phi)F^{\leftharpoonup}_{x'})]\\ &- s(\phi)g[\alpha^-(-s(\phi)F^{\leftharpoonup}_{y'} + c(\phi)F^{\rightharpoonup}_{x'}) + \alpha^+(-s(\phi)F^{\rightharpoonup}_{y'} + c(\phi)F^{\leftharpoonup}_{x'})]\Big\}\end{aligned} \tag{55}$$

$$\begin{aligned}\frac{\partial F^{\rightharpoonup}_{y'}}{\partial t} = -(c_1 g - \Omega)F^{\rightharpoonup}_{x'} + c_1\Big\{ -\frac{\partial F^{\rightharpoonup}_{y'}}{\partial z} &+ s(\phi)\frac{\partial}{\partial z}[\alpha^-(-s(\phi)F^{\leftharpoonup}_{y'} + c(\phi)F^{\rightharpoonup}_{x'}) + \alpha^+(-s(\phi)F^{\rightharpoonup}_{y'} + c(\phi)F^{\leftharpoonup}_{x'})]\\ &- c(\phi)g[\alpha^+(-s(\phi)F^{\leftharpoonup}_{y'} + c(\phi)F^{\rightharpoonup}_{x'}) + \alpha^-(-s(\phi)F^{\rightharpoonup}_{y'} + c(\phi)F^{\leftharpoonup}_{x'})]\Big\}\end{aligned} \tag{56}$$

$$\begin{aligned}\frac{\partial F^{\leftharpoonup}_{x'}}{\partial t} = -(c_1 g + \Omega)F^{\leftharpoonup}_{y'} + c_1\Big\{ +\frac{\partial F^{\leftharpoonup}_{x'}}{\partial z} &+ c(\phi)\frac{\partial}{\partial z}[\alpha^-(-s(\phi)F^{\leftharpoonup}_{y'} + c(\phi)F^{\rightharpoonup}_{x'}) + \alpha^+(-s(\phi)F^{\rightharpoonup}_{y'} + c(\phi)F^{\leftharpoonup}_{x'})]\\ &+ s(\phi)g[\alpha^+(-s(\phi)F^{\leftharpoonup}_{y'} + c(\phi)F^{\rightharpoonup}_{x'}) + \alpha^-(-s(\phi)F^{\rightharpoonup}_{y'} + c(\phi)F^{\leftharpoonup}_{x'})]\Big\}\end{aligned} \tag{57}$$

$$\begin{aligned}\frac{\partial F^{\leftharpoonup}_{y'}}{\partial t} = +(c_1 g + \Omega)F^{\leftharpoonup}_{x'} + c_1\Big\{ +\frac{\partial F^{\leftharpoonup}_{y'}}{\partial z} &- s(\phi)\frac{\partial}{\partial z}[\bar{\alpha}^+(-s(\phi)F^{\leftharpoonup}_{y'} + c(\phi)F^{\rightharpoonup}_{x'}) + \bar{\alpha}^-(-s(\phi)F^{\rightharpoonup}_{y'} + c(\phi)F^{\leftharpoonup}_{x'})]\\ &+ c(\phi)g[\alpha^-(-s(\phi)F^{\leftharpoonup}_{y'} + c(\phi)F^{\rightharpoonup}_{x'}) + \alpha^+(-s(\phi)F^{\rightharpoonup}_{y'} + c(\phi)F^{\leftharpoonup}_{x'})]\Big\}\end{aligned} \tag{58}$$

Inserting Bloch wave solutions: $\Psi = e^{i(kz-\omega t)}\sum_n \psi_n e^{(2n-1)i(gz-\Omega t)}$ yields (defining $\omega_n = \omega + (2n-1)\Omega$ and $k_n = k + (2n-1)g$):

$$
\begin{aligned}
\omega_n F^{\rightharpoonup}_{x'\,n} = +i(c_1 g - \Omega)F^{\rightharpoonup}_{y'\,n} - c_1\Big\{ &- k_n F^{\rightharpoonup}_{x'\,n} - k_n c(\phi)[\bar{\alpha}^{+}(-s(\phi)F^{\leftharpoonup}_{y'\,n} + c(\phi)F^{\rightharpoonup}_{x'\,n}) + \bar{\alpha}^{-}(-s(\phi)F^{\rightharpoonup}_{y'\,n} + c(\phi)F^{\leftharpoonup}_{x'\,n})] \\
&+ igs(\phi)[\bar{\alpha}^{-}(-s(\phi)F^{\leftharpoonup}_{y'\,n} + c(\phi)F^{\rightharpoonup}_{x'\,n}) + \bar{\alpha}^{+}(-s(\phi)F^{\rightharpoonup}_{y'\,n} + c(\phi)F^{\leftharpoonup}_{x'\,n})]\Big\}
\end{aligned}
\tag{59}
$$

$$
\begin{aligned}
\omega_n F^{\rightharpoonup}_{y'\,n} = -i(c_1 g - \Omega)F^{\rightharpoonup}_{x'\,n} - c_1\Big\{ &- k_n F^{\rightharpoonup}_{y'\,n} + k_n s(\phi)[\bar{\alpha}^{-}(-s(\phi)F^{\leftharpoonup}_{y'\,n} + c(\phi)F^{\rightharpoonup}_{x'\,n}) + \bar{\alpha}^{+}(-s(\phi)F^{\rightharpoonup}_{y'\,n} + c(\phi)F^{\leftharpoonup}_{x'\,n})] \\
&+ igc(\phi)[\bar{\alpha}^{+}(-s(\phi)F^{\leftharpoonup}_{y'\,n} + c(\phi)F^{\rightharpoonup}_{x'\,n}) + \bar{\alpha}^{-}(-s(\phi)F^{\rightharpoonup}_{y'\,n} + c(\phi)F^{\leftharpoonup}_{x'\,n})]\Big\}
\end{aligned}
\tag{60}
$$

$$
\begin{aligned}
\omega_n F^{\leftharpoonup}_{x'\,n} = -i(c_1 g + \Omega)F^{\leftharpoonup}_{y'\,n} - c_1\Big\{ &+ k_n F^{\leftharpoonup}_{x'\,n} + k_n c(\phi)[\bar{\alpha}^{-}(-s(\phi)F^{\leftharpoonup}_{y'\,n} + c(\phi)F^{\rightharpoonup}_{x'\,n}) + \bar{\alpha}^{+}(-s(\phi)F^{\rightharpoonup}_{y'\,n} + c(\phi)F^{\leftharpoonup}_{x'\,n})] \\
&- igs(\phi)[\bar{\alpha}^{+}(-s(\phi)F^{\leftharpoonup}_{y'\,n} + c(\phi)F^{\rightharpoonup}_{x'\,n}) + \bar{\alpha}^{-}(-s(\phi)F^{\rightharpoonup}_{y'\,n} + c(\phi)F^{\leftharpoonup}_{x'\,n})]
\end{aligned}
\tag{61}
$$

$$
\begin{aligned}
\omega_n F^{\leftharpoonup}_{y'\,n} = +i(c_1 g + \Omega)F^{\leftharpoonup}_{x'\,n} - c_1\Big\{ &+ k_n F^{\leftharpoonup}_{y'\,n} - k_n s(\phi)[\bar{\alpha}^{+}(-s(\phi)F^{\leftharpoonup}_{y'\,n} + c(\phi)F^{\rightharpoonup}_{x'\,n}) + \bar{\alpha}^{-}(-s(\phi)F^{\rightharpoonup}_{y'\,n} + c(\phi)F^{\leftharpoonup}_{x'\,n})] \\
&- igc(\phi)[\bar{\alpha}^{-}(-s(\phi)F^{\leftharpoonup}_{y'\,n} + c(\phi)F^{\rightharpoonup}_{x'\,n}) + \bar{\alpha}^{+}(-s(\phi)F^{\rightharpoonup}_{y'\,n} + c(\phi)F^{\leftharpoonup}_{x'\,n})]\Big\}
\end{aligned}
\tag{62}
$$

Abbreviating the notation further with $s = s(\phi)$, $c = c(\phi)$, $\bar{g} = c_1 g$ and $\bar{k}_n = c_1 k_n$ we can rewrite the latter as a standard eigenvalue problem:

$$
\omega_n \begin{pmatrix} \mathbf{F}^{\rightharpoonup}_n \\ \mathbf{F}^{\leftharpoonup}_n \end{pmatrix} = \begin{pmatrix} \mathbb{M}^{\rightharpoonup}_{\rightharpoonup n} & \mathbb{M}^{\rightharpoonup}_{\leftharpoonup n} \\ \mathbb{M}^{\leftharpoonup}_{\rightharpoonup n} & \mathbb{M}^{\leftharpoonup}_{\leftharpoonup n} \end{pmatrix} \begin{pmatrix} \mathbf{F}^{\rightharpoonup}_n \\ \mathbf{F}^{\leftharpoonup}_n \end{pmatrix}
\tag{63}
$$

where the four matrices couple the forward and backward propagating states $\mathbf{F}^{\rightharpoonup}_n = (F^{\rightharpoonup}_{x'\,n}; F^{\rightharpoonup}_{y'\,n})$ and $\mathbf{F}^{\leftharpoonup}_n = (F^{\leftharpoonup}_{x'\,n}; F^{\leftharpoonup}_{y'\,n})$, and read:

$$
\mathbb{M}^{\rightharpoonup}_{\rightharpoonup n} = \begin{pmatrix} \bar{k}_n + c(\phi)[\bar{\alpha}^{+}c(\phi)\bar{k}_n - i\bar{\alpha}^{-}s(\phi)\bar{g}] & +i(\bar{g}-\Omega) + s(\phi)[-\bar{\alpha}^{-}c(\phi)\bar{k}_n + i\bar{\alpha}^{+}s(\phi)\bar{g}] \\ -i(\bar{g}-\Omega) + c(\phi)[-\bar{\alpha}^{-}s(\phi)\bar{k}_n - i\bar{\alpha}^{+}c(\phi)\bar{g}] & \bar{k}_n + s(\phi)[\bar{\alpha}^{+}s(\phi)\bar{k}_n + i\bar{\alpha}^{-}c(\phi)\bar{g}] \end{pmatrix}
\tag{64}
$$

$$
\mathbb{M}^{\rightharpoonup}_{\leftharpoonup n} = \begin{pmatrix} c(\phi)[\bar{\alpha}^{-}c(\phi)\bar{k}_n - i\bar{\alpha}^{+}s(\phi)\bar{g}] & s(\phi)[-\bar{\alpha}^{+}c(\phi)\bar{k}_n + i\bar{\alpha}^{-}s(\phi)\bar{g}] \\ c(\phi)[-\bar{\alpha}^{+}s(\phi)\bar{k}_n - i\bar{\alpha}^{-}c(\phi)\bar{g}] & s(\phi)[\bar{\alpha}^{-}s(\phi)\bar{k}_n + i\bar{\alpha}^{+}c(\phi)\bar{g}] \end{pmatrix}
\tag{65}
$$

$$
\mathbb{M}^{\leftharpoonup}_{\rightharpoonup n} = \begin{pmatrix} c(\phi)[-\bar{\alpha}^{-}c(\phi)\bar{k}_n + i\bar{\alpha}^{+}s(\phi)\bar{g}] & s(\phi)[\bar{\alpha}^{+}c(\phi)\bar{k}_n - i\bar{\alpha}^{-}s(\phi)\bar{g}] \\ c(\phi)[\bar{\alpha}^{+}s(\phi)\bar{k}_n + i\bar{\alpha}^{-}c(\phi)\bar{g}] & s(\phi)[-\bar{\alpha}^{-}s(\phi)\bar{k}_n - i\bar{\alpha}^{+}c(\phi)\bar{g}] \end{pmatrix}
\tag{66}
$$

$$
\mathbb{M}^{\leftharpoonup}_{\leftharpoonup n} = \begin{pmatrix} -\bar{k}_n + c(\phi)[-\bar{\alpha}^{+}c(\phi)\bar{k}_n + i\bar{\alpha}^{-}s(\phi)\bar{g}] & -i(\Omega+\bar{g}) + s(\phi)[\bar{\alpha}^{-}c(\phi)\bar{k}_n - i\bar{\alpha}^{+}s(\phi)\bar{g}] \\ +i(\Omega+\bar{g}) + c(\phi)[\bar{\alpha}^{-}s(\phi)\bar{k}_n + i\bar{\alpha}^{+}c(\phi)\bar{g}] & -\bar{k}_n + s(\phi)[-\bar{\alpha}^{+}s(\phi)\bar{k}_n - i\bar{\alpha}^{-}c(\phi)\bar{g}] \end{pmatrix}
\tag{67}
$$

Note that one peculiarity of this solution strategy is that the ordering of the bands is modified as a result of the coupling between harmonics inherent to the screwing basis vectors. As a result, diagonalizing Eq. 63 yields all the eigenvalues, but not in increasing order.

Supplementary Note 2 - Change of basis in matrix form

Having derived the correct basis which uncouples the bands, we can rewrite the change of basis in a more compact matrix form $\mathbb{F} = \hat{S}\mathbb{G}$ as:

$$
\begin{pmatrix} F^{\rightharpoonup}_{x'} \\ F^{\rightharpoonup}_{y'} \\ F^{\leftharpoonup}_{x'} \\ F^{\leftharpoonup}_{y'} \end{pmatrix} = \begin{pmatrix} c(\theta) & c(\theta) & s(\theta) & s(\theta) \\ -s(\theta) & -s(\theta) & c(\theta) & c(\theta) \\ c(\theta) & -c(\theta) & s(\theta) & -s(\theta) \\ -s(\theta) & s(\theta) & c(\theta) & -c(\theta) \end{pmatrix} \begin{pmatrix} D_x \\ \bar{B}_y \\ D_y \\ -\bar{B}_x \end{pmatrix}
\tag{68}
$$

Since the matrix above is orthogonal, its inverse corresponds to its transpose, and we can write immediately:

$$
\begin{pmatrix} D_x \\ \bar{B}_y \\ D_y \\ -\bar{B}_x \end{pmatrix} = \begin{pmatrix} c(\theta) & -s(\theta) & c(\theta) & -s(\theta) \\ c(\theta) & -s(\theta) & -c(\theta) & s(\theta) \\ s(\theta) & c(\theta) & s(\theta) & c(\theta) \\ s(\theta) & c(\theta) & -s(\theta) & -c(\theta) \end{pmatrix} \begin{pmatrix} F^{\rightharpoonup}_{x'} \\ F^{\rightharpoonup}_{y'} \\ F^{\leftharpoonup}_{x'} \\ F^{\leftharpoonup}_{y'} \end{pmatrix}
\tag{69}
$$

Thus, we have:

$$D_x = c(\theta)F_{x'}^{\rightharpoonup} - s(\theta)F_{y'}^{\rightharpoonup} + c(\theta)F_{x'}^{\leftharpoondown} - s(\theta)F_{y'}^{\leftharpoondown} \qquad D_y = s(\theta)F_{x'}^{\rightharpoonup} + c(\theta)F_{y'}^{\rightharpoonup} + s(\theta)F_{x'}^{\leftharpoondown} + c(\theta)F_{y'}^{\leftharpoondown} \tag{70}$$

$$\bar{B}_y = c(\theta)F_{x'}^{\rightharpoonup} - s(\theta)F_{y'}^{\rightharpoonup} - c(\theta)F_{x'}^{\leftharpoondown} + s(\theta)F_{y'}^{\leftharpoondown} \qquad -\bar{B}_x = s(\theta)F_{x'}^{\rightharpoonup} + c(\theta)F_{y'}^{\rightharpoonup} - s(\theta)F_{x'}^{\leftharpoondown} - c(\theta)F_{y'}^{\leftharpoondown} \tag{71}$$

Supplementary Note 2 - Numerical Floquet-Bloch Calculations

Here we solve for $k(\omega)$, in terms of the E and H fields. Note that, due to a dual symmetry between space and time we can to solve for $\omega(k)$ and for with the same sets of equations following the substitutions:

$$D \leftrightarrow E \qquad B \leftrightarrow H \qquad \alpha_{\varepsilon/\mu} \leftrightarrow \bar{\alpha}_{\varepsilon/\mu} = -\frac{\alpha_{\varepsilon/\mu}}{1+2\alpha_{\varepsilon/\mu}} \qquad k \leftrightarrow \omega \qquad g \leftrightarrow \Omega \tag{72}$$

Since we are going to use E, H as our variables, it is useful to write down the time derivatives of the material tensors:

$$\frac{\partial \varepsilon}{\partial t} = 2\varepsilon_1\alpha_\varepsilon\Omega \begin{pmatrix} s(2\theta^-) & -c(2\theta^-) & 0 \\ -c(2\theta^-) & -s(2\theta^-) & 0 \\ 0 & 0 & 0 \end{pmatrix} \qquad \frac{\partial \mu}{\partial t} = 2\mu_1\alpha_\mu\Omega \begin{pmatrix} -s(2\theta^+) & c(2\theta^+) & 0 \\ c(2\theta^+) & s(2\theta^+) & 0 \\ 0 & 0 & 0 \end{pmatrix} \tag{73}$$

so that Maxwell's Equations give us, for normal incidence:

$$\frac{1}{\mu_1}\left(\frac{\partial E_z}{\partial y} - \frac{\partial E_y}{\partial z}\right) = +2\alpha_\mu\Omega(s(2\theta^+)H_x - c(2\theta^+)H_y) - (1+2\alpha_\mu s^2(\theta^+))\frac{\partial H_x}{\partial t} + 2\alpha_\mu s(\theta^+)c(\theta^+)\frac{\partial H_y}{\partial t} \tag{74}$$

$$-\frac{1}{\mu_1}\left(\frac{\partial E_z}{\partial x} - \frac{\partial E_x}{\partial z}\right) = +2\alpha_\mu\Omega(-c(2\theta^+)H_x - s(2\theta^+)H_y) + 2\alpha_\mu s(\theta^+)c(\theta^+)\frac{\partial H_x}{\partial t} - (1+2\alpha_\mu c^2(\theta^+))\frac{\partial H_y}{\partial t} \tag{75}$$

$$\frac{1}{\varepsilon_1}\left(\frac{\partial H_z}{\partial y} - \frac{\partial H_y}{\partial z}\right) = 2\alpha_\varepsilon\Omega(s(2\theta^-)E_x - c(2\theta^-)E_y) + (1+2\alpha_\varepsilon c^2(\theta^-))\frac{\partial E_x}{\partial t} + 2\alpha_\varepsilon s(\theta^-)c(\theta^-)\frac{\partial E_y}{\partial t} \tag{76}$$

$$-\frac{1}{\varepsilon_1}\left(\frac{\partial H_z}{\partial x} - \frac{\partial H_x}{\partial z}\right) = 2\alpha_\varepsilon\Omega(-c(2\theta^-)E_x - s(2\theta^-)E_y) + 2\alpha_\varepsilon s(\theta^-)c(\theta^-)\frac{\partial E_x}{\partial t} + (1+2\alpha_\varepsilon s^2(\theta^-))\frac{\partial E_y}{\partial t} \tag{77}$$

and using double-angle formulae:

$$\frac{1}{\mu_1}\left(\frac{\partial E_z}{\partial y} - \frac{\partial E_y}{\partial z}\right) = +2\alpha_\mu\Omega(s(2\theta^+)H_x - c(2\theta^+)H_y) - [1+\alpha_\mu(1-c(2\theta^+))]\frac{\partial H_x}{\partial t} + \alpha_\mu s(2\theta^+)\frac{\partial H_y}{\partial t} \tag{78}$$

$$-\frac{1}{\mu_1}\left(\frac{\partial E_z}{\partial x} - \frac{\partial E_x}{\partial z}\right) = +2\alpha_\mu\Omega(-c(2\theta^+)H_x - s(2\theta^+)H_y) + \alpha_\mu s(2\theta^+)\frac{\partial H_x}{\partial t} - [1+\alpha_\mu(1+c(2\theta^+))]\frac{\partial H_y}{\partial t} \tag{79}$$

$$\frac{1}{\varepsilon_1}\left(\frac{\partial H_z}{\partial y} - \frac{\partial H_y}{\partial z}\right) = +2\alpha_\varepsilon\Omega(s(2\theta^-)E_x - c(2\theta^-)E_y) + [1+\alpha_\varepsilon(1+c(2\theta^-))]\frac{\partial E_x}{\partial t} + \alpha_\varepsilon s(2\theta^-)\frac{\partial E_y}{\partial t} \tag{80}$$

$$-\frac{1}{\varepsilon_1}\left(\frac{\partial H_z}{\partial x} - \frac{\partial H_x}{\partial z}\right) = +2\alpha_\varepsilon\Omega(-c(2\theta^-)E_x - s(2\theta^-)E_y) + \alpha_\varepsilon s(2\theta^-)\frac{\partial E_x}{\partial t} + [1+\alpha_\varepsilon(1-c(2\theta^-))]\frac{\partial E_y}{\partial t} \tag{81}$$

We now assume ansatz of the form: $\psi \sim e^{i(kz-\omega t)}\sum_n \psi_n e^{2in(gz-\Omega t)}$, so that the respective derivatives yield:

$$\frac{\partial \psi}{\partial t} = -ie^{i(kz-\omega t)}\sum_n(\omega+2n\Omega)\psi_n e^{2in(gz-\Omega t)} \tag{82}$$

$$\frac{\partial \psi}{\partial z} = ie^{i(kz-\omega t)}\sum_n(k+2ng)\psi_n e^{2in(gz-\Omega t)} \tag{83}$$

We can thus write Maxwell's equations as:

$$\frac{\partial E_y}{\partial z} = \mu_1\left[2\alpha_\mu\Omega(-s(2\theta^+)H_x + c(2\theta^+)H_y) - \alpha_\mu s(2\theta^+)\frac{\partial H_y}{\partial t} + [1+\alpha_\mu(1-c(2\theta^+))]\frac{\partial H_x}{\partial t}\right] \tag{84}$$

$$\frac{\partial E_x}{\partial z} = \mu_1\left[2\alpha_\mu\Omega(-c(2\theta^+)H_x - s(2\theta^+)H_y) + \alpha_\mu s(2\theta^+)\frac{\partial H_x}{\partial t} - [1+\alpha_\mu(1+c(2\theta^+))]\frac{\partial H_y}{\partial t}\right] \tag{85}$$

$$\frac{\partial H_y}{\partial z} = \varepsilon_1\left[2\alpha_\varepsilon\Omega(-s(2\theta^-)E_x + c(2\theta^-)E_y) - \alpha_\varepsilon s(2\theta^-)\frac{\partial E_y}{\partial t} - [1+\alpha_\varepsilon(1+c(2\theta^-))]\frac{\partial E_x}{\partial t}\right] \tag{86}$$

$$\frac{\partial H_x}{\partial z} = \varepsilon_1\left[2\alpha_\varepsilon\Omega(-c(2\theta^-)E_x - s(2\theta^-)E_y) + \alpha_\varepsilon s(2\theta^-)\frac{\partial E_x}{\partial t} + [1+\alpha_\varepsilon(1-c(2\theta^-))]\frac{\partial E_y}{\partial t}\right] \tag{87}$$

so that substituting in the fields yields the eigenvalue problem for k:

$$(k+2n'g)E_{y,n'} = \sum_n \Big\{ \alpha_\mu \Omega[(\delta_{n',n+1}-\delta_{n',n-1})H_{x,n} - i(\delta_{n',n+1}+\delta_{n',n-1})H_{y,n}] \tag{88}$$

$$- \frac{i}{2}\alpha_\mu(\omega+2n\Omega)(\delta_{n',n+1}-\delta_{n',n-1})H_{y,n} \tag{89}$$

$$- (1+\alpha_\mu)(\omega+2n\Omega)\delta_{n',n}H_{x,n} + \frac{1}{2}\alpha_\mu(\omega+2n\Omega)(\delta_{n',n+1}+\delta_{n',n-1})H_{x,n}\Big\}$$

$$(k+2n'g)E_{x,n'} = \sum_n \Big\{ \alpha_\mu \Omega[+(\delta_{n',n+2}-\delta_{n',n-1})H_{y,n} + i(\delta_{n',n+1}+\delta_{n',n-1})H_{x,n}] \tag{90}$$

$$+ \frac{i}{2}\alpha_\mu(\omega+2n\Omega)(\delta_{n',n+1}-\delta_{n',n-1})H_{x,n} \tag{91}$$

$$+ (1+\alpha_\mu)(\omega+2n\Omega)\delta_{n',n}H_{y,n} + \frac{1}{2}\alpha_\mu(\omega+2n\Omega)(\delta_{n',n+1}+\delta_{n',n-1})H_{y,n}\Big\}$$

$$(k+2n'g)H_{y,n'} = \sum_n \Big\{ \alpha_\varepsilon \Omega[+(\delta_{n',n+1}-\delta_{n',n-1})E_{x,n} - i(\delta_{n',n+1}+\delta_{n',n-1})E_{y,n}] \tag{92}$$

$$- \frac{i}{2}\alpha_\varepsilon(\omega+2n\Omega)(\delta_{n',n+1}-\delta_{n',n-1})E_{y,n} \tag{93}$$

$$+ (1+\alpha_\varepsilon)(\omega+2n\Omega)\delta_{n',n}E_{x,n} + \frac{1}{2}\alpha_\varepsilon(\omega+2n\Omega)(\delta_{n',n+1}+\delta_{n',n-1})E_{x,n}\Big\}$$

$$(k+2n'g)H_{x,n'} = \sum_n \Big\{ \alpha_\varepsilon \Omega[+(\delta_{n',n+1}-\delta_{n',n-1})E_{y,n} + i(\delta_{n',n+1}+\delta_{n',n-1})E_{x,n}] \tag{94}$$

$$+ \frac{i}{2}\alpha_\varepsilon(\omega+2n\Omega)(\delta_{n',n+1}-\delta_{n',n-1})E_{x,n} \tag{95}$$

$$- (1+\alpha_\mu)(\omega+2n\Omega)\delta_{n',n}E_{y,n} + \frac{1}{2}\alpha_\varepsilon(\omega+2n\Omega)(\delta_{n',n+1}+\delta_{n',n-1})E_{y,n}\Big\}$$

In matrix form, we can write this system as:

$$k_n \begin{pmatrix} \mathbf{E}_y \\ \mathbf{E}_x \\ \mathbf{H}_y \\ \mathbf{H}_x \end{pmatrix} = \begin{pmatrix} \mathbb{M}^{E_y}_{E_y} & 0 & \mathbb{M}^{E_y}_{H_y} & \mathbb{M}^{E_y}_{H_x} \\ 0 & \mathbb{M}^{E_x}_{E_x} & \mathbb{M}^{E_x}_{H_y} & \mathbb{M}^{E_x}_{H_x} \\ \mathbb{M}^{H_y}_{E_y} & \mathbb{M}^{H_y}_{E_x} & \mathbb{M}^{H_y}_{H_y} & 0 \\ \mathbb{M}^{H_x}_{E_y} & \mathbb{M}^{H_x}_{E_x} & 0 & \mathbb{M}^{E_x}_{E_x} \end{pmatrix} \begin{pmatrix} \mathbf{E}_y \\ \mathbf{E}_x \\ \mathbf{H}_y \\ \mathbf{H}_x \end{pmatrix} \tag{96}$$

where the respective $\mathbb{M}$ matrices are tridiagonal, and read:

$$(M^{E_y}_{E_y})_{n',n} = -2ng\delta_{n,n'} \tag{97}$$
$$(M^{E_x}_{E_x})_{n',n} = -2ng\delta_{n,n'} \tag{98}$$
$$(M^{H_y}_{H_y})_{n',n} = -2ng\delta_{n,n'} \tag{99}$$
$$(M^{H_x}_{H_x})_{n',n} = -2ng\delta_{n,n'} \tag{100}$$
$$(M^{E_y}_{H_y})_{n',n} = -\frac{k_y k_x}{\omega + 2n\Omega}\delta_{n',n} + i\alpha_\mu\Omega(\delta_{n',n+1} + \delta_{n',n-1}) + i\frac{\alpha_\mu}{2}(\omega + 2n\Omega)(\delta_{n',n+1} - \delta_{n',n-1}) \tag{101}$$
$$(M^{E_y}_{H_x})_{n',n} = \frac{k_y^2}{\omega + 2n\Omega}\delta_{n',n} - \alpha_\mu\Omega(\delta_{n',n+1} - \delta_{n',n-1}) - (1+\alpha_\mu)(\omega + 2n\Omega)\delta_{n,n'} - \frac{1}{2}\alpha_\mu(\omega + 2n\Omega)(\delta_{n',n+1} + \delta_{n',n-1}) \tag{102}$$
$$(M^{E_x}_{H_y})_{n',n} = -\frac{k_x^2}{\omega + 2n\Omega}\delta_{n',n} - \alpha_\mu\Omega(\delta_{n',n+1} - \delta_{n',n-1}) + (1-\alpha_\mu)(\omega + 2n\Omega)\delta_{n,n'} - \frac{1}{2}\alpha_\mu(\omega + 2n\Omega)(\delta_{n',n+1} + \delta_{n',n-1}) \tag{103}$$
$$(M^{E_x}_{H_x})_{n',n} = \frac{k_x k_y}{\omega + 2n\Omega}\delta_{n',n} - i\alpha_\mu\Omega(\delta_{n',n+1} + \delta_{n',n-1}) - i\frac{\alpha_\mu}{2}(\omega + 2n\Omega)(\delta_{n',n+1} - \delta_{n',n-1}) \tag{104}$$
$$(M^{H_y}_{E_y})_{n',n} = \frac{k_x k_y}{\omega + 2n\Omega}\delta_{n',n} - i\alpha_\varepsilon\Omega(\delta_{n',n+1} + \delta_{n',n-1}) + i\frac{\alpha_\varepsilon}{2}(\omega + 2n\Omega)(\delta_{n',n+1} - \delta_{n',n-1}) \tag{105}$$
$$(M^{H_y}_{E_x})_{n',n} = -\frac{k_y^2}{\omega + 2n\Omega}\delta_{n',n} + \alpha_\varepsilon\Omega(\delta_{n',n+1} - \delta_{n',n-1}) + (1-\alpha_\varepsilon)(\omega + 2n\Omega)\delta_{n,n'} + \frac{1}{2}\alpha_\varepsilon(\omega + 2n\Omega)(\delta_{n',n+1} + \delta_{n',n-1}) \tag{106}$$
$$(M^{H_x}_{E_y})_{n',n} = \frac{k_x^2}{\omega + 2n\Omega}\delta_{n',n} + \alpha_\varepsilon\Omega(\delta_{n',n+1} - \delta_{n',n-1}) - (1+\alpha_\mu)(\omega + 2n\Omega)\delta_{n,n'} + \frac{1}{2}\alpha_\varepsilon(\omega + 2n\Omega)(\delta_{n',n+1} + \delta_{n',n-1}) \tag{107}$$
$$(M^{H_x}_{E_x})_{n',n} = -\frac{k_x k_y}{\omega + 2n\Omega}\delta_{n',n} + i\alpha_\varepsilon\Omega(\delta_{n',n+1} + \delta_{n',n-1}) - i\frac{\alpha_\varepsilon}{2}(\omega + 2n\Omega)(\delta_{n',n+1} - \delta_{n',n-1}) \tag{108}$$

Supplementary Note 4 - Transmission through Archimedes' Screw

In order to calculate transmission through a finite-length Archimedes' screw we expand the E and H fields into a Floquet-Bloch basis and calculate the wavevector eigenvalues k for a fixed frequency of the impinging wave. Let us assume solutions of the form $\mathbf{E} = E_x\mathbf{x} + E_y\mathbf{y}$, $\mathbf{H} = H_x\mathbf{x} + H_y\mathbf{y}$, where the eigen-solutions for each field are of the form:

$$\psi = e^{i(kx-\omega t)}\sum_n \Phi_n e^{2in(gx-\Omega t)} \tag{109}$$

In the absence of modulation, the vacuum eigenvalues read, for both x and y polarisations:

$$k^{\pm}_{vn} = -2ng \pm (\omega + 2n\Omega), \tag{110}$$

and the corresponding eigenvectors are

$$\mathbf{v}^{\pm}_{\mathbf{n,x}} = \begin{pmatrix}0\\1\\1\\0\end{pmatrix}/\sqrt{2} \qquad \mathbf{v}^{\pm}_{\mathbf{n,y}} = \begin{pmatrix}1\\0\\0\\-1\end{pmatrix}/\sqrt{2} \tag{111}$$

.

Subsequently, we can write the fields at the left (1) and right (2) interfaces of the metamaterial as superpositions of the vacuum eigenvectors:

$$\begin{pmatrix}\mathbf{E^{(1)}_{v,z}}\\\mathbf{H^{(1)}_{v,y}}\end{pmatrix} = \mathbf{M^{vinc}e^{(1)}_{vinc}} + \mathbf{M^{vref}e^{(1)}_{vref}} \qquad \begin{pmatrix}\mathbf{E^{(2)}_{v,z}}\\\mathbf{H^{(2)}_{v,y}}\end{pmatrix} = \mathbf{M^{vinc}e^{(2)}_{vtra}} \tag{112}$$

and inside the metamaterial:

$$\begin{pmatrix}\mathbf{E^{(1)}_{m,z}}\\\mathbf{H^{(1)}_{m,y}}\end{pmatrix} = \mathbf{M^{m}e_{m}} \qquad \begin{pmatrix}\mathbf{E^{(2)}_{m,z}}\\\mathbf{H^{(2)}_{m,y}}\end{pmatrix} = \mathbf{M^{m}Pe_{m}} \tag{113}$$

where $\mathbf{M^{vinc}}$ and $\mathbf{M^{vref}}$ are rectangular matrices containing the right- and left-propagating vacuum eigenvectors respectively, $\mathbf{M^{m}}$ is a square matrix containing all eigenvectors inside of the metamaterial, and $\mathbf{P}$ is a diagonal matrix

$P_{mn} = \exp{(ik_m d)}\delta_{mn}$ which propagates each eigenvector from the left to the right interface. The vector $\mathbf{e_{v_inc}}$ contains the amplitudes of the input fields at the left interface, whereas the vectors $\mathbf{e_{v_ref}}$ and $\mathbf{e_{v_tra}}$ contain the unknown reflected and transmitted amplitudes respectively. Applying the continuity of E_z and H_y at the two interfaces, we arrive at a matrix equation:

$$\begin{pmatrix} \mathbf{A} & \mathbf{B} \end{pmatrix} \begin{pmatrix} \mathbf{e^{(2)}_{vtr}} \\ \mathbf{e^{(2)}_{vref}} \end{pmatrix} = \begin{pmatrix} \mathbf{M^{vinc}} & 0 \end{pmatrix} \begin{pmatrix} \mathbf{e^{(1)}_{vinc}} \\ 0 \end{pmatrix} \tag{114}$$

where:

$$\mathbf{A} = \mathbf{M^{m}}(\mathbf{M}^{m}\mathbf{P})^{-1}\mathbf{M^{vinc}} \qquad \mathbf{B} = -\mathbf{M^{mvref}} \tag{115}$$

are rectangular matrices, such that their concatenation is square, so that the transmitted and reflected amplitudes can be readily calculated by inverting Eq. 114.
